# Global smoking-related deaths averted due to MPOWER policies implemented at the highest level between 2007 and 2020

**DOI:** 10.1186/s12992-023-01012-w

**Published:** 2024-05-07

**Authors:** Greg Lyle, Delia Hendrie

**Affiliations:** Curtin University School of Population Health, Perth, Bentley Campus, 6102, Australia

**Keywords:** Global health, Smoking cessation, Prevention, Effectiveness, Deaths, Inequality, Public policy

## Abstract

**Background:**

In response to the harm caused by tobacco use worldwide, the World Health Organization (WHO) World Health Assembly actioned the WHO Framework Convention on Tobacco Control (FCTC) in 2005. To help countries meet their FCTC obligations, the WHO introduced in 2008 the MPOWER policy package and by 2020 the FCTC had been ratified by 182 parties. The package consists of six evidence-based demand reduction smoking cessation policies to assist countries to achieve best practice. We used published evaluation results and replicated the published model to estimate current policy achievement and demonstrate the impact and equity of the MPOWER policy package in reducing the global number of smokers and smoking-attributable deaths (SADs) between 2007 and 2020.

**Methods:**

We replicated an evaluation model (the Abridged SimSmoke model) used previously for country impact assessments and validated our replicated reduction in SADs for policies between 2014 and 2016 against the published results. The replicated model was then applied to report on the country level SADs averted from achieving the highest level of implementation, that is best practice in MPOWER policies, between 2016 and 2020. The latest results were then combined with past published results to estimate the reduction in SADs since the commencement of the MPOWER policy package. Country level income status was used to investigate the equity in the uptake of MPOWER policies worldwide.

**Results:**

Identical estimates for SADs in 41 out of 56 MPOWER policies implemented in 43 countries suggested good agreement in the model replication. The replicated model overestimated the reduction in SADs by 159,800 (1.5%) out of a total of 10.5 million SADs with three countries contributing to the majority of this replication discrepancy. Updated analysis estimated a reduction of 8.57 million smokers and 3.37 million SADs between 2016 and 2020. Between 2007 and 2020, 136 countries had adopted and maintained at least one MPOWER policy at the highest level which was associated with a reduction in 81.0 million smokers and 28.3 million SADs. Seventy five percent of this reduction was in middle income countries, 20% in high income and less than 5% in low income countries.

**Conclusions:**

Considerable progress has been made by MPOWER policies to reduce the prevalence of smokers globally. However, there is inequality in the implementation and maintenance, reach and influence, and the number of SADs averted. Future research to modify the model could provide a more comprehensive evaluation of past and future progress in tobacco control policies, worldwide.

## Introduction

Tobacco is a leading risk factor for morbidity and mortality globally [1]. The number of smokers increased from 721 million in 1980 to 967 million in 2012 [2] and tobacco attributable deaths were estimated to be 5.4 million in 2005 and projected to be 8.3 million by 2030 [3]. In response to the harm caused by tobacco use worldwide, the World Health Assembly, the decision-making body of the World Health Organisation (WHO) in 2005 actioned the WHO Framework Convention on Tobacco Control (FCTC). The FCTC is a global evidenced-based public health treaty ratified in 2020 by 182 parties, 181 individual countries represented by 32 low, 98 middle and 51 high income countries and the European Union, equating to 90% of the world’s population [4].

Ratified countries continually implement the treaty’s articles to reaffirm the right of all people to a high standard of health [5]. The treaty has enabled the mobilisation of a global tobacco control movement through international cooperation and information exchange and by supporting an evidence-based legal framework to overcome challenges to tobacco control measures by the tobacco industry and others [6]. At the national level, the FCTC provides an agenda for action and a tool for governments to plan and implement their tobacco control work moving from a restricted health focus to recognition of the engagement between, and broader responsibilities of, different government sectors in controlling tobacco use [7].

To help meet the FCTC obligations, the WHO, introduced the MPOWER policy package in 2008. The MPOWER policy package provides strategic guidelines that assist in intensifying the efforts to promote smoking cessation policies [8]. The package includes six demand reduction articles reflected as polices and comprising of; M – Monitor tobacco use and prevention policies, P – Protect people from tobacco smoke, O – Offer help to quit tobacco use, W – Warn about the dangers of tobacco, E – Enforce bans on tobacco advertising, promotion and sponsorship and R – Raise taxes on tobacco [4]. The WHO reports on the summary indicators of country achievements for each of the POWER policies, assigning values from 1 to 5, and 1 to 4 for the M policy. Implementation of a policy at the level of best practice (highest value) reflects the most effective way to reduce tobacco use. Further information on the package is available in the technical note as part of the WHO reporting [4].

Worldwide it is estimated that 1.18 billion people regularly smoke tobacco causing seven million deaths in 2020 [9]. There is large variation in smoking prevalence between countries reflecting their uptake of tobacco use and MPOWER tobacco control interventions. For example, Dai et al. [9] investigated the evolution of the global smoking epidemic over the past half century. They reported considerable variation in male and female age-standardised prevalence for 2020 showing that percentages ranged from less than 10% to over 40% in some countries. Considerable success in FCTC implementation exists in Europe with P and R being the most implemented interventions, however, this varies between countries [10]. Successful implementation of P interventions in South America has made it the first sub-region of the Americas to establish 100% smoke-free environments [11]. Moderate improvements in tobacco control have been undertaken in the South East Asian [12] and Eastern Mediterranean regions with M and W interventions the most implemented [13, 14]. In South Asian, most FCTC articles have been neglected or addressed in a discordant way with key barriers being the lack of public awareness of smoking harms and the benefits of quitting, poor implementation of anti-smoking laws, and socio-cultural acceptance of tobacco use resulting in a continued increase in smoking prevalence [15].

A narrative review of 128 studies reported that the FCTC has resulted in significant gains in tobacco control where rapid MPOWER implementation and progress to best practice was consistently the most effective strategies to encourage quitting, reduce tobacco consumption and prevalence, and tobacco-related health risks [16]. Since the ratification of the FCTC there has been significant gains in the passing of bans on smoking in pubs and indoor offices (P) [17, 18] and placing health warnings on packaging (W) [18] as well as bans on direct advertising (E) [17]. Globally, the time since the establishment of the FCTC had suggestive positive associations with bans on sponsorship and point of sale advertising (E) but negative associations between increasing taxes and the time since FCTC ratification (R) [18].

Evaluation of the MPOWER package has shown global reductions in smoking prevalence and cigarette consumption and increased quit attempts [19–22]. For 126 countries (36% high income, 50% middle-income and 13% low-income countries), the association between reaching the highest level of implementation of MPOWER policies between 2007 and 2014 and smoking prevalence between 2005 and 2015 was found to decrease average smoking prevalence from 24.7% in 2005 to 22.2% in 2015 [19]. Reaching the highest level for each additional policy was associated with a decrease in smoking prevalence of 0.94 percentage points and an average relative decrease of 3.2% in smoking prevalence after controlling for geographical subregion, income level and WHO FCTC party status [19]. To assess the effectiveness of the implementation of the whole MPOWER package worldwide, two studies used a composite score approach where individual component scores for each of the six MPOWER policies were summed for each country and year, and associated with smoking prevalence. A one unit composite score increase reduced smoking prevalence by 0.2 percentage points among adults [23]. Those countries with higher initial tobacco control efforts and higher smoking burden reduced their smoking prevalence rates by 0.39 to 0.50 percentage points with a unit score increase [24].

Focus on the evaluation of countries implementing individual policies worldwide has shown different impacts on smoking prevalence and cessation rates. For example, a study using data from 2009 to 2017 found that a one-unit increment in MPOWER achievement score for each of the P, W, E and R interventions was associated with a 1%, 2.1%, 1.8% and 0.7% decrease in global smoking prevalence, respectively [25]. There has been limited analysis of the impact of the M policy as it is not seen as a demand reduction measure. However, an early 2008 study, using a composite score for assessing MPOWER implementation reported that a one unit increase in the M score value reduced smoking prevalence by about one percentage point [20]. Additionally, a one-unit increase for the R policy resulted in a 0.95 and 0.41 percentage point decrease in smoking prevalence for males and females [20].

Variation also exists in the effectiveness of the MPOWER policies. Worldwide analysis from 2007 to 2014 has shown that the display of graphic health warnings on packages (W) has been associated with 0.9–3% decrease in adult smoking prevalence [26]. Systematic reviews on the worldwide impact of implementing the W policy has shown an increase in quit calls and attempts, and perceptions of health risks and reductions in smoking behaviour [27, 28]. Analysis of data from 2007 to 2014 for 63 countries reported that point of sale advertising bans (E) have been associated with a 0.7% decrease in adult smoking prevalence [29]. Economic data from Austria between 1997 and 2015 concluded that for the R policy a 1% increase in tobacco price resulted in 0.7% decrease in tobacco consumption per person [30]. Finally, individual measures within these policies have also been evaluated for their effectiveness to increase cessation rates. A meta-analysis found that measures within the P policy such as bans on smoking in hospitals and in workplaces were estimated to produce a 16% and 44% increase in cessation rates, respectively [31]. Efforts within the O policy such as behavioural interventions towards society and by telephone, cessation services provided by health professional and pharmacological intervention reported 27%, 47% and 83% increases in cessation rates. Use of anti-smoking television advertising and media campaigns (E), and regulations on cigarette packaging (W) reported 15% and 12% reduction in smoking prevalence [31].

The progress in implementing demand reduction cessation policies to 2020 has led to up to five billion lives [32] or around 65% of the world population [17] being covered by at least one measure at best practice while 4.4 billion people or 98 countries are covered by at least two MPOWER measures [4]. The evidence for adoption, progress and effectiveness is compelling but the adoption of the full MPOWER package at the highest level has been relatively slow with less than 0.5% of the world’s population in 2019 being covered [33].

The policy impact of the MPOWER policies implemented at a level of best practice has also been evaluated in terms of reducing the number of smokers and smoking-attributable deaths (SADs). Of significance, are several studies using the Abridged SimSmoke model to estimate these impacts across the years since inception [34–36]. These studies estimate that globally, 7.4 million SADs were saved from adopting the highest level of MPOWER measures between 2007 and 2010 [34]. Revision of these estimates through to 2014 reported that 22 million SADs were saved [35] while those implemented from 2014 to 2016 identified a further 14.6 million fewer SADs [36].

This aim of this study was to follow and build on the evidence base of other studies [34, 35] in the systematic estimation of the effectiveness of recent and continued progress in tobacco control using the SADs outcome measure. Replicating the model incorporating newer data will allow for updated comparisons within and between countries on the reach and influence of singular policies and the package as a whole. An updated time series will also highlight gaps in the equity of adoption and achievement, globally. We firstly, replicated the Abridged SimSmoke model used in the global evaluation of MPOWER policies [34, 37]. This model is a cut down version of the SimSmoke tobacco policy control model which has been extensively validated [36] and because of its numerous iterations in evaluating the impact of the MPOWER policies represents the current gold standard in evaluation of tobacco control policy. The model has been fully documented allowing for replicability and ease of reproducibility, furthermore, the multiple applications provide addition confidence in the effect sizes used. Where the methodology was unclear, assumptions were made and documented. Secondly, we tested how robust our replication of the model was by validating the replicated results against the 2014–2016 evaluation [36] in order to determine the predictive capacity of the model’s replication. Thirdly, the replicated model, with no adaptations made after validation, and the latest biennial WHO report on the global tobacco epidemic was used to estimate SADs from country progress in reaching the highest level of MPOWER policies between 2016 and 2020. The results from our current estimates and those from previous published evaluations [34–36] were combined to provide estimates for the reduction in SADs since the introduction of the MPOWER package in 2007 through to 2020. Lastly, we determined the degree of equity in the implementation and maintenance of the MPOWER policies to reduce SADs worldwide over the 2007–2020 time period.

## Methods

### The Abridged SimSmoke model

The Abridged SimSmoke tobacco control policy model [34, 36, 37] calculates the country level SADs averted since the introduction of a MPOWER policy implemented at the highest level, referred to as best practice. The model is based on (i) WHO monitoring data (ii) the total number of smokers affected, (iii) the effect size parameters for incremental policy change derived from the SimSmoke tobacco control policy model; (iv) additional adjustment factors to reflect country level variation in health awareness, urbanisation, compliance, the extent of publicity and to estimate longer-term effects; and (v) the assumption based that a proportion of all regular cigarette smokers will eventually be killed by their habit [38, 39]. We replicated the model as best as possible from the methodology outlined from the latest studies [36, 37]. Where the methodology was unclear, assumptions were made based on the data and these are outlined below. We validated the updated model against published evaluations for 2014 to 2016 [36], then applied the model to new data for 2016 to 2020.

### Collating WHO monitoring data by country

Countries reaching the best practice of an MPOWER policy were collated from the WHO’s monitoring data [4]. Policy levels reported in 2014, before the best practice was achieved, and in 2016, were noted to determine the degree of incremental policy change to 2016. This change determined the magnitude of the effect size applied in the validation of the model. In applying the replicated model, new data was collected for the evaluation of MPOWER policies between 2016 and 2020. The changes to 2020 were confirmed by representatives of the WHO Tobacco Free Initiative before evaluation. As in other evaluations [34], because it is not seen as a demand reduction measure, the monitoring (M) policy was excluded in the evaluation for comparability.

### Calculating the number of smokers affected by country

To calculate the number of smokers affected, male and female populations, aged 15 and over, and sex-specific smoking prevalence rates by country were extracted from online data repositories [4, 40, 41].

### Policy effect sizes

#### Introduction

Levy et al. [37] report that the effect of the incremental policy change to best practice in MPOWER policies was evaluated through the relative long-term percentage reduction (effect size) in smoking prevalence from pre-policy implementation. Long-term effect size was reported as the policy change after 40 years and derived by multiplying a policy specific short-term effect size and policy specific adjustors by the percentage change in smoking prevalence in the first year of implementation. Short-term effect sizes were taken from the internationally validated SimSmoke model [42, 43] and reproduced in the Abridged Sim Smoke model [36, 37] for each policy measure based on policy evaluation studies in high-income countries (HICs). For all policies, a long-term multiplier reflecting the ratio of the relative change in prevalence after 40 years to the relative change in short-term prevalence in the first year was applied to transform the short-term to a long-term effect size. For the POWE policies, an awareness (or knowledge) adjustor was applied based on whether the country is a low- or middle-income country (LMICs) classified by their standing in the World Bank’s income rankings in the year of analysis that they achieved the best practice level [44]. The income level was used to reflect the country’s likely stage in the tobacco epidemic with the awareness adjustor reflecting the potential to affect attitudes and awareness at earlier stages in the tobacco epidemic [37]. For example, in the model the effects of the health warnings (W) policy are doubled in LMICs due to the lower initial level of awareness or knowledge. For P and O policies, the effect sizes taken from HICs were adjusted by the level of urbanisation to reflect the ability of these policies to reach and influence the population [34, 37]. Furthermore, for the P policy, a country level labour force adjustment was also included to reflect that smoke-free work site laws primarily influence the population who work indoors [45]. Additionally, for P and E policies, the effect sizes were adjusted by a level of country compliance assessed by national experts who reported a value between 0 (minimum) and 10 (maximum) for compliance to smoke free laws and banning of tobacco advertising, promotion and sponsorship (TAPS) [4]. None of the above adjustments were made for the R policy, however, adjustments were made for smoking prevalence price elasticities reflecting whether the country was categorised as HIC or LMIC. These values are presented in Appendix 1.

#### Calculation of the long-term effect sizes of specific MPOWER policies.

##### P Policy: Smoke free air laws

Country level achievements in smoke free laws were assessed by the WHO on the number of places covered by these laws and their compliance. Reaching the best practice of P requires smoking bans to be placed in five indoor workplaces, indoor restaurants, pubs and bars and other indoor places [4].

**Assumptions used in deriving short-term effect sizes from WHO monitoring data**

The short-term effect size of banning smoking in all public places in the model is additive with effect sizes for specific places outlined in Appendix 1. We found that the method of assigning the short-term effect size for the five indoor workplaces was unclear [37], therefore based short-term effect sizes on the assumption that the 6% was allocated proportionately according to the number of changes to have occurred in the five indoor workplaces. For example, if bans were in place for three indoor workplaces in 2016 increasing to all indoor workplaces in 2020, a short-term effect size of 2.4% was applied based on 0.4 ratio (2/5) of 6%.

**Model equation and assumptions**

The calculation for long-term effect size (*LT-SFA*_*i*_) is written in Eq. 1 which includes policy specific adjustments based on country characteristics. The level of urbanisation was represented as one minus the percentage employed in agriculture (*Urban*_*i*_). Levy et al. [37] state that an adjustor for labour force participation based on one minus the unemployment rate was included, however, this appeared to be missing from their mathematical appendix [37]. Given these adjustor values by country were outlined in the methodology [37], and that the adjustor reflects a reduction in reach of the policies, we have included an adjustor in Eq. 1 as *Labour*_*i*_.

Levy et al. [37] made the assumption that half of the bans’ influence will occur when the law is passed, the other half will depend on country specific compliance and publicity. It should be noted that Levy et al. use the term “enforcement”, whereas the WHO use “compliance”. For consistency we have adopted the semantic terminology of “compliance” as per the WHO data reporting in Eq. 1 since this is where the data used in the equation originates from. Within their effect size table Levy et al. [37] compliance was described as a “Ranking out of 10 converted to a percent”. In the text however, the compliance index was described as a ratio with the compliance value scaled from 0 to 1. We followed the method outlined in the text and used the average of compliance values between the two time periods (Comp_i_,_SFA_). Where no compliance values were reported a value of 2 (law implemented but with low compliance) was assigned as per Levy et al. [37]. When one value was only reported we used this singular value instead of the average.

Publicity levels values (Pub_i_) are based the amount of expenditure on tobacco control per capita (in US dollars and 15 years plus population). Two versions of publicity level values multipliers were reported in Levy et al. [37] based on the low (< US$0.1), medium (US$0.1-US$2) and high (> US$2) per capita income. We used the multipliers 0.5, 0.75 and 1, respectively, as they were reported in the supplementary material mathematical appendix, in their effect size table and in the first MPOWER evaluation [34]. Where no values were reported a multiplier value of 0.5 was used.

1 $$LT-SFA_{i} = ST-SFA_{i} * LT-Mult_{SFA}* Aware_{i,SFA} * Urban_{i} *Labour_{i}*[0.5* (1+Comp_{i,SFA}*Pub_{i})]$$

where *LT-SFA*_*i*_ is the long-term effect size for a country (*i*) implementing smoke-free air laws (*SFA*), *ST-SFA*_*i*_ is the short-term effect size of *SFA* policies implemented, *LT-Mult*_*SFA*_ is the long-term multiplier, *Aware*_*i*_*,*_*SFA*_ is the awareness multiplier, *Urban*_*i*_ is urbanisation level, *Labour*_*i*_ is labour participation, *Comp*_*i*_*,*_*SFA*_ is the compliance multiplier and *Pub*_*i*_ is the publicity multiplier.

##### O Policy: Cessation services

The treatment for tobacco dependence requires the provision of behavioural and pharmacological cessation services. Reaching the highest level for offering cessation services means the country has a national quit line available and both nicotine replacement therapy (NRT) and some cessation services are to be cost-covered within five health sectors [4].

**Assumptions used in deriving short-term effect sizes from WHO monitoring data**

All quit lines that were available were assumed to be operating active [37]. Provision of cost coverage of NRT is required to reach the highest level. However, this is not considered within the model. Instead, we followed the model’s assumption that was based on NRT availability in a general store or pharmacy either with or without a prescription (Rx) [34, 37] which may or may not have costs covered. The WHO definition of providing “some cessation services to be cost covered” to achieve the highest level is difficult to quantify. In Levy et al.’s effect size table assignment is by availability only [37]. We followed the model’s assumption based on availability only and assumed the same effect for those sectors that reported that the provision of treatments was “partial” or “fully” cost covered. To reflect availability and the differing proportions that can be implemented across the provision of treatment services in the five sectors we used the proportional allocation method described in Sect. P Policy: Smoke free air laws to allocate effect sizes. For example, if the provision of treatments were not available in any sectors in 2016 and increased to three sectors in 2020, a short-term effect size of 1.35% was applied based on a 0.6 ratio (3/5) of 2.25%.

An additional requirement in attributing effect size was that the programs were well publicised with Levy et al. [37] stating the inclusion of a publicity adjustor, however no adjustor was included in their mathematical equations. Given publicity’s influence on program uptake we have included the publicity adjustor, as in Eq. 1, in Eq. 2.

**Model equation and assumptions**

The calculation for long-term effect size (*LT-CTP*_*i*_) is written in Eq. 2.

2 $$LT-CTP_{i} = ST-CTP_{i} * LT-Mult_{CTP} * Aware_{i,CTP} *Urban_{i},*Pub_{i}$$

where *LT-CTP*_*i*_ is the long-term effect size for a country (*i*) implementing cessation treatment policies (CTP), *ST-CTP*_*i*_ is the short-term effect size of *CTP* policies implemented, *LT-Mult*_*CTP*_ is the long-term multiplier, *Aware*_*i*_*,*_*CTP*_ is the awareness multiplier, *Urban*_*i,*_is the urbanisation level, and *Pub*_*i*_ is the publicity level.

##### W Policy: Health warnings

Assessment is made on whether a country includes specific health warning information on cigarette packaging. Warning information on the package are graded on the size, content and characteristics of warnings on the package’s surface (front and back). Reaching the highest level requires large warnings with all appropriate characteristics [4].

**Assumptions used in deriving short-term effect sizes from WHO monitoring data**

Information on bans were operationalised by the WHO to grade in ascending order each country’s law within a scale from 1 to 5. These were: Data not reported (coded as 1), No warning or small warnings (2), Medium size warnings missing some or many appropriate characteristics or large warnings missing many appropriate characteristics (3), Medium size warnings with all appropriate characteristics or large warnings missing some appropriate characteristics (4), Large warnings with all appropriate characteristics (5) [4].

Within the model the effect sizes used account for the magnitude of change, from the previous level of health warnings to achieve the complete level. Levy et al. [37] table the effect sizes into three categories; complete, medium and low health warnings. These category labels do not match the operationalisation of the indicator which has five categories. We assumed that countries attaining the complete level who previously were deemed by the WHO to have a score of 2 are given the full effect size of 4%. Moving from a score of 3 or 4 to a complete level (5) is given an effect size of 3% and 2%, respectively, reflecting the change in effect size to the complete level (4%) (Appendix 1).

**Model equation and assumptions**

The calculation for long-term effect size (*LT-HW*_*i*_) is written in Eq. 3.

3 $$LT-HW_{i} = ST-HW_{i} * LT-Mult_{HW} * Aware_{i,HW,}$$

where *LT-HW*_*i*_ is the long-term effect size for a country (*i*) implementing health warnings policies (*HW*), *ST-HW*_*i*_ is the short-term effect size of *HW* policies implemented, *LT-Mult*_*HW*_ is the long-term multiplier, and *Aware*_*i*_*,*_*HW*_ is the awareness multiplier.

##### E Policy: Bans on tobacco advertising, promotion and sponsorship (TAPS)

Country level achievements were assessed based on the degree to which bans are placed and their level of compliance. The highest level was achieved with bans (at least 90% of the population covered) on all forms of direct and indirect advertising [4].

**Assumptions used in deriving short-term effect sizes from WHO monitoring data**

Information on bans were operationalised by the WHO to grade in ascending order each country’s law within a scale from 1 to 5. These were: Data not reported (coded as 1), Complete absence of ban, or ban that does not cover national television, radio and print media (2), Ban on national television, radio and print media only (3), Ban on national television, radio and print media as well as on some but not all other forms of direct and/or indirect advertising (4) and Ban on all forms of direct and/or indirect advertising (or at least 90% of the population covered by complete subnational bans)(5) [4].

Within the model the effect sizes used accounted for the magnitude of change, from the previous level of bans to achieve the complete level. Levy et al. [37] table the effect sizes into three categories; complete, strong and partial bans on what they term TAPS as marketing. These category labels do not match the operationalisation of the indicator which has five categories. We assumed that countries attaining the complete level who previously were deemed by the WHO to have a score of 2 are given the full effect size of 5%. Moving from a score of 3 or 4 to a score of 5 is given an effect size of 4% and 2%, respectively, reflecting the change in effect size to the complete level (5%) (Appendix 1). We followed the method outlined in Sect. P Policy: Smoke free air laws to assign a value for the level of compliance.

**Model equation and assumptions**

The calculation for long-term effect size (*LT-BTAPS*_*i*_) is written in Eq. 3. Levy et al. [37] posit that a total lack of compliance may reduce the impact by half.

4 $$LT-BTAPS_{i} = ST-BTAPS_{i} * LT-Mult_{BTAPS} * Aware_{i,BTAPS} *0.5 (1+Comp_{i,BTAPS})$$

where *LT-BTAPS*_*i*_ is the long-term effect size for a country (*i*) implementing bans on tobacco advertising, promotion and sponsorship (*BTAPS*), *ST-BTAPS*_*i*_ is the short-term effect size of *BTAPS* policies implemented, *LT-Mult*_*BTAPS*_ is the long-term multiplier, *Aware*_*i*_*,*_*BTAPS*_ is the awareness multiplier, and *Comp*_*i,BTAPS*_ is the compliance level.

##### R Policy: Raising cigarette taxes

Countries were grouped according to the percentage contribution of all tobacco taxes to the retail price of a pack of 20 of the most popular brand of cigarettes. Retail prices in local currency were graded as (1) < 25% of retail price is tax, (2) ≥ 25% and < 50% of retail price is tax, (3) ≥ 50% and < 75% of retail price is tax, (4) ≥ 75% of retail price is tax. Countries are identified by the WHO as reaching the highest level when total taxes, including excise and value added taxes are ≥ 75% of retail price [4].

**Assumptions used in deriving short-term effect sizes from WHO monitoring data**

Those countries that reach the highest level of percentage contribution of tobacco taxes were identified. The calculation of effect size was based on the change in inflation-adjusted prices. This requires the collection of each countries nominal pack prices reported by the WHO and inflation rates between two time periods. For example, in the replication of model, the latest price represented the price in 2016 and the past price was the price in 2014. The latest nominal pack price was adjusted by inflation to reflect the real price in the past time period’s currency. When the real price was equal to or less than the nominal pack price in the past time period, a zero effect size was assigned.

**Model equation and assumptions**

The latest nominal pack price is adjusted by the cumulated inflation rate (*Cum_Inflation*_*Y1, Y2*_) (Eq. 5) to make it comparable, in real terms, to the past price (*Real_Price *_*Y2*_) (Eq. 6) to identify the influence of the tax change on price.

5 $$Cum\_Inflation_{Y1, Y2} = (1+Inf_{Y1}/100)*(1+ Inf_{Y2}/100)$$

where *Cum_Inflation*_*Y1, Y2*_ is the cumulated inflation rate (*Inf*) between past (*Y1*) and latest (*Y2*).

6 $$Real\_Price_{Y2} = (1/Cum\_Inflation_{Y1, Y2}*Price_{Y2})$$

where *Real_Price*_*Y2*_ is the adjusted price in for the latest year (*Y2*) in past (*Y1*) price terms, *Cum_Inflation*_*Y1, Y2*_ is the cumulated inflation rate between the past (*Y1*) and latest (*Y2*) years , and *Price*_*Y2*_ is the nominal price in the latest year (*Y2*).

The average price of the past (*Y1*) and latest (*Y2*) is calculated (*Average_Price*_*Y1,Y2*_) (Eq. 7) and is used in calculating the relative price change between both years (*Rel_Price_Change*_*Y1,Y2*_) (Eq. 8).

7 $$Average\_Price_{Y1,Y2}=(Price_{Y1}+Real\_Price_{Y2})/2$$

where *Average_Price*_*Y1,Y2*_ is the average of the past price (*Y1*) and the latest adjusted price (*Y2*) in past price (*Y1*) dollars (*Real_Price*_*Y2*_).

8 $$Rel\_Price\_Change_{Y1,Y2} = (Price_{Y1} - Real\_Price_{Y2})/ Average\_price_{Y1,Y2}$$

where *Rel_Price_Change*_*Y1,Y2*_ is the relative price change between the past (*Y1*) and latest (*Y2*) prices.

Long-term effect size (*LT-RT*_*i*_) (Eq. 9) is calculated as the relative price change by the smoking prevalence price elasticities (0.15 for HICs and 0.20 for LMICs) and a long-term multiplier (*LT-Mult*_*RT*_).

9 $$LT-RT_{i}= Rel\_Price\_Change_{Y1,Y2} * Price\_Elast*100*LT Mult_{RT}$$

where *LT-RT*_*i*_ is the long-term effect size, *Price_Elast* is the price elasticities of 0.15 for HICs or 0.2 for LMICs, and *LT-Mult*_*RT*_ is the long-term multiplier.

### Calculation of the reduction in the number of smokers

Long-term effect sizes from each policy implemented at the best practice level for each country were applied to the total number of smokers affected. This calculation estimated the total reduction in the number of smokers.

### Calculation of the reduction in smoking-attributable deaths (SADs)

The number of SADs averted was calculated following the assumption that 50% of these smokers will die from smoking in HICs, with an additional adjustment for a lower relative mortality risk of smoking in LMICs (0.65) [37]. Lower and Upper bounds of SADs were reported as ± 50% to reflect the estimates. Due to the limited precision of prevalence rate and populations, all projected number of smokers and deaths were rounded to three significant digits to avoid false precision [37].

### Validation of the replicated Abridged SimSmoke model against the published results

We replicated the calculation of the long-term effect sizes for each MPOWER policy by country who reached the best practice level for the period between 2014 and 2016 to estimate the number of SADs averted. Three measures were used to determine our ability to replicate the model from the published documentation which assessed the impact of any modifications made and to judge the robustness of its predictive capability at the country, policy and package scales. Firstly, effect sizes and number of SADs from our replicated model were compared against the 2014–2016 published results [36] to determine where finer level country differences were apparent within and across each MPOWER policy. Secondly, to determine the overall predictive accuracy and the robustness of the replicated model, the country level estimates of the number of SADs averted by policy and the package as a whole were summed and compared to published estimates.

### Determining the SADs averted by reaching the highest level of MPOWER policy between 2016 and 2020

We applied the replicated model to predict the SADs averted due to achieving best practice from data published by the WHO [4] for MPOWER policies between 2016 and 2020.

### Determining the SADs averted by reaching the highest level of MPOWER policies between 2007 and 2020

Country level estimates of the reduction in SADs from earlier evaluations of the three time periods between 2007–2016 [34–36] were collected and the SADs for LMICs were adjusted to reflect the lower relative mortality risk of smoking in LMICs. These past estimates were collated along with those estimated in this study. We reviewed past and current WHO data reporting on the year when each country met the best practice level for MPOWER policies to ensure that there were no overlapping time periods. Countries that achieved the highest level in earlier years but were no longer at the highest level for a policy in 2020 were excluded. Countries that achieved the highest level twice, due to dropping to a lower level after achieving the highest level, before again achieving the highest level, were only included the second time when the highest level was achieved to avoid double counting. Reduction in SADs was then presented by MPOWER policy and time period to evaluate the progress and the potential lives saved of policy implementation.

### Determining the SADs averted by reaching the highest level of MPOWER policies between 2007 and 2020 by low, middle and high income countries

We assigned the reduction in country level SADs calculated in Sect. Determining the SADs averted by reaching the highest level of MPOWER policies between 2007 and 2020 to their World Bank’s income ranking in the year of analysis that they achieved the best practice level [44]. Country income status was classified as low income (LIC), middle income (MIC) and high income (HIC). Where countries moved rankings over time while achieving a best practice policy, we took the income status at the time of the policy change for the calculation of total smokers, reduction in smokers and SADs, as this was taken from past studies. This occurred in three countries; Pakistan moving from LIC to MIC and Latvia and Croatia moving from MIC to HIC status. For reporting the number of countries which achieved best practice income status at the time of the last policy change was used.

We aggregated the total of smokers affected, the reduction in smokers and the number of SADs averted by country income status to qualify the reach and influence of the MPOWER policies and the equity of their implementation and maintenance, worldwide.

## Results

### Comparison of the outputs from the replicated Abridged SimSmoke model with the published estimates

Comparison of results from the replicated model with the results published by Levy et al. [36, 37] produced 159,800 additional SADs across the P, O, and E measures of the MPOWER policies (Table 1). This equates to an overestimation of 1.5% out of a total of 10.5 million SADs averted previously estimated between 2014 and 2016. Effect sizes and the number of SADs were replicated exactly for W and R policies. The largest difference between the results estimated in the replicated model and those published by Levy et al. [36, 37] was due to the inclusion of a quit line in India. This measure was not included within the O policy in Levy’s analysis and subsequently increased the total by 249,000 SADs. This value was offset mostly by the underestimation of effect sizes and SADs for other countries adopting the P and E policies. This was especially the case for Romania which made up 78% of the underestimation of SADs for the P policy and for Afghanistan which made up 62% of the underestimation of SADs for the E policy. For P policies, four out of the six countries had a difference of less than 1,000 SADs, but the replicated results for the E policy were less accurate with five out of the seven countries having an underestimated difference of more than 1,000 SADs. Overall, within each MPOWER policy, identical SADs were obtained in 41 out of 56 MPOWER policies implemented in 43 countries. Underprediction of SADs occurred in 11 countries while over prediction occurred in 4 countries, both of varying magnitudes (Table 1).

**Table 1:** Comparison of long-term effect sizes and reduction in smoking-attributable deaths (SADs): package, policy and country-specific results

Intervention/ Country	Long-term effect size		Reduction in SADS		Difference in reduction of SADs (1)-(2) (%)
	**Replication: Approach %**	**Levy 2020%**	**Replication: Approach (1)**	**Levy 2020 (2)**	
***Smoke-free Air laws (P)***					
Afghanistan	0.8	1.5	8,700	16,200	-7,500 (18)
Cambodia	1.3	1.3	7,790	7,930	-140 (0.3)
El Salvador	8.0	8.7	11,800	12,700	-900 (2)
Laos	2.5	2.7	10,400	11,300	-900 (2)
Romania	7.6	9.5	126,000	158,000	-32,000 (78)
Uganda	2.2	2.1	14,700	13,800	900 (2)
***Total Smoke-free Air laws (P)***^***d***^			*179,000*	*220,000*	*-41,000 (19)*
***Cessation services (O)***					
El Salvador	0.0	0.0	0	0	0
Estonia	0.0	0.0	0	79	-79 (0.03)
India	0.75	0.0	249,000	0	249,000 (99.6)
Jamaica	0.0	0.0	0	0	0
Luxembourg	2.2	0.7	1,230	405	825
Senegal	0.6	0.6	1,340	1,280	60
***Total Cessation services (O)***^***d***^			*251,600*	*1,760*	*250,000*
***Health warning (W)***^***b***^					
Armenia	8.0	8.0	15,000	15,000	0
Austria	6.0	6.0	67,800	67,800	0
Bangladesh	8.0	8.0	673,000	673,000	0
Belarus	8.0	8.0	56,500	56,500	0
Belgium	4.0	4.0	53,400	53,400	0
Bulgaria	12.0	12.0	91,600	91,600	0
Burkina Faso^cb^	8.0	8.0	31,400	31,400	0
Cambodia	12.0	12.0	71,900	71,900	0
Chad^cb^	12.0	12.0	27,900	27,900	0
Czech Republic	6.0	6.0	92,900	92,900	0
Denmark	4.0	4.0	18,700	18,700	0
Estonia	6.0	6.0	10,600	10,600	0
Finland	6.0	6.0	28,600	28,600	0
France	4.0	4.0	344,000	344,000	0
Germany	6.0	6.0	656,000	656,000	0
Greece	6.0	6.0	127,000	127,000	0
Hungary	4.0	4.0	52,200	52,200	0
India	16.0	16.0	5,700,000	5,700,000	0
Ireland	4.0	4.0	18,400	18,400	0
Italy	6.0	6.0	367,000	367,000	0
Laos	12.0	12.0	50,000	50,000	0
Latvia	4.0	4.0	12,700	12,700	0
Lithuania	6.0	6.0	22,200	22,200	0
Malta	4.0	4.0	1,890	1,890	0
Moldova	12.0	12.0	32,400	32,400	0
Netherlands	6.0	6.0	111,000	111,000	0
Poland	6.0	6.0	280,000	280,000	0
Portugal	6.0	6.0	61,700	61,700	0
Romania^a c^	8.0	8.0	132,000	132,000	0
Senegal^cb^	16.0	16.0	35,600	35,600	0
Suriname^cb^	8.0	8.0	2,700	2,700	0
Slovakia	6.0	6.0	41,700	41,700	0
Sweden	6.0	6.0	47,100	47,100	0
UK	4.0	4.0	248,000	248,000	0
***Total Health warning (W)***^***d***^			*9,600,000*	*9,600,000*	*0*
***Bans on tobacco advertising, promotion and sponsorship (TAPS) (E)***					
Afghanistan	4.4	7.2	47,900	78,500	-30,600 (62)
Kuwait	1.3	1.3	4,450	4,450	0
Nigeria	6.5	6.5	121,000	121,000	0
Qatar	2.5	3.0	5,430	6,490	-1,060 (2)
Moldova	3.9	4.9	10,500	13,300	-2,800 (6)
Senegal	2.6	8.5	5,790	18,800	-13,010 (27)
Uganda	6.5	6.5	43,300	44,400	-1,100 (2)
***Total (TAPS) (E)***^***d***^			*238,000*	*287,000*	*-49,000 (17)*
***Raising taxes (R)***					
Argentina	16.7	16.7	392,000	392,000	0
Austria	0.1	0.1	1,100	1,100	0
Malta	2.3	2.3	1,070	1,070	0
***Total Raising taxes (R)***^***d***^			*394,000*	*394,000*	*0*
***Total MPOWER polices ***^***e***^			**10,662,600**	**10,502,800**	**159,800 (1.5)**

^a^Levy et al. [36] report (Table 1) that the long-term effect size was 8.0%,however, within the supplementary material [37] this was reported as 4.0% and used a country total number of smokers affected of 12,383,023 rather than 5,090,000

^b^Included in Levy. et al. [36] Table 1 but not in supplementary material [37]

^c^We used the reduction in SADs in Levy et al. [36] Table 1 and adjusted the number of SADs for LMICs using the adjustor (0.65) as reported in this article’s supplementary material [37]

^d^Rounding was applied to all estimates as in Levy et al. [37]

^e^Calculated as the difference between total for all policies in the replication approach and Levy et al. [36] rather than the sum of the differences

### Estimated smoking-attributable deaths (SADs) averted between 2016 and 2020

A total of 75 MPOWER policies were implemented at best practice level in 58 countries, with 45 countries achieving best practice in one policy and 13 in more than one policy. Ethiopia, Guyana, Jordan and Saudi Arabia achieved this highest level in three MPOWER policies. Approximately 236 million smokers were affected by these policy changes, which were projected to lead to 8.57 million fewer smokers and a reduction in 3.37 million SADs (Table 2). Uncertainty in the estimates for SADs averted is reflected in the wide range (1.68 to 5.05 million). Achieving the highest-level health warnings (W) accounted for the greatest share (69%) of SADs averted, followed by bans on TAPS (E) (13%), cessation services (O) (8%), raising taxes (R) (7%) and smoke-free air laws (P) (3%). Of the 11 countries adopting smoke-free air laws (P), Jordan (39%), Ethiopia (18%) and Bolivia (17%) made up 74% of the reduction in SADs (Appendix 2). Ten countries adopted cessation services (O) policies with the Philippines making up 65% of the reduction in SADs (Appendix 3). While Austria, Sweden and Tonga achieved best practice, we calculated 0% effect sizes for their incremental policy changes from the data reported. Twenty-four countries adopted health warnings (W) at best practice on their country’s cigarette packaging. The populated countries of the United States of America (45%) and Pakistan (23%) made up 68% of the reduction in SADs (Appendix 4). It should be noted that the United Staes have not ratified the FCTC but have been included by the WHO in their reporting. Sixteen countries adopted bans on TAPS (E) with Venezuela and Algeria accounting for 24% and 12% of the reduction in SADs (Appendix 5). Fourteen countries achieved the highest level of raising cigarette taxes (R) with Georgia (26%), the Netherlands (24%), Denmark (16%) contributing 66% of the reduction in SADs (Appendix 6). After adjusting for inflation, we found that the real price fell for four countries (Brazil, Morocco, Portugal and Thailand) resulting in no long-term effect sizes being applied.

**Table 2:** Reduction in smoking-attributable deaths (SADs) from countries reaching the highest level by MPOWER package and policies between 2016 and 2020^1^

Policy measure	Total number of smokers affected (column %)	Proportional reduction in number of smokers (row %)	Proportional reduction in SADs			
			**HIC risks**^**2**^** (column %)**	**LMIC adjusted **^**3**^** (column %)**	**Lower bound **^**4**^	**Upper bound **^**4**^
P-Smoke-free air laws	7,760,000 (3)	325,000 (4)	163,000 (4)	106,000 (3)	53,000	158,000
O-Cessation services	31,800,000 (13)	718,000 (2)	360,000 (8)	260,000 (8)	130,000	389,000
W-Health warnings	102,000,000 (43)	5,600,000 (5)	2,800,000 (65)	2,320,000 (69)	1,160,000	3,480,000
E- Bans on TAPS	31,300,000 (13)	1,310,000 (4)	653,000 (15)	442,000 (13)	221,000	663,000
R-Raising taxes	63,100,000 (27)	615,000 (1)	308,000 (7)	241,000 (7)	120,000	361,000
**All policies**	**236,000,000**	**8,570,000 (4)**	**4,280,000**	**3,370,000**	**1,680,000**	**5,050,000**

^1^Rounding has been applied to all estimates as in Levy et al. [37]

^2^The High Income Country (HIC) risks apply the 0.5 multiplier to the reduction in total number of smokers in all countries to reflect that 50% of all people who smoke will die from smoking. This column applies the HIC multiplier to all countries

^3^The Low and Middle Income Country (LMIC) adjusted estimates apply the 0.65 multiplier to LMICs countries to reflect the lower mortality risk of smoking in these countries. This column applies the risk of death according to a country’s income level (HIC or LMIC)

^4^Applied to LMIC adjusted estimates; Lower and Upper bounds set at ± 50% of SADs

### Impact of countries achieving the highest-level MPOWER policies: 2007–2020

There was a total of 233 MPOWER policies implemented and maintained at best practice level in 136 countries (countries can maintain multiple policies) between 2007 and 2020 (Table 3). One country (Turkey) maintained all MPOWER policies (*n* = 5 policies) at the highest level, 18 countries have achieved and maintained three MPOWER polices (*n* = 54 policies), 57 countries achieved and maintained two policies (*n* = 114 policies), and 60 countries have achieved and maintained one MPOWER policy (*n* = 60 policies). Brazil had only three policies evaluated as best practice because the remaining two policies had achieved best practice in advance to the 2007 WHO baseline evaluation period for MPOWER policies. Other countries with similar achievements before 2007 were also not incorporated into the analysis. The number of countries implementing and maintaining policies varied, with the highest being for the W policy and lowest for O and R (Table 3). A total of 22 countries (9% LIC, 59% MIC, 32% HIC) did not maintain their achievement and were removed from the analysis (data not shown). Specifically, 4 countries (all MIC) were removed from P policy analysis, nine from the O (55% HIC) and nine from the R policy analysis were removed (66% MIC).

**Table 3:** Reduction in smoking-attributable deaths (SADs) for countries reaching the highest level by MPOWER package and policies: 2007–2020^1^

MPOWER policies	Years	Countries	Total number of smokers affected	Total reduction in number of smokers^3^	Reduction in SADs^3^	
					**HIC risks **^**4**^	**LMIC-adjusted **^**5**^
**P-Smoke-free air laws**	2007–2010	19 (36)	85,400,000 (40)	3,390,000 (23)	1,700,000 (23)	1,200,000 (24)
	2010–2014	17 (32)	104,000,000 (49)	10,400,000 (70)	5,210,000 (70)	3,380,000 (69)
	2014–2016	6 (11)	14,100,000(7)	676,000 (5)	338,000 (5)	220,000 (4)
	2016–2020	11 (21)	7,760,000 (4)	325,000 (2)	163,000 (2)	106,000 (2)
	**Total: 2007–2020**	**53 (39)**	**211,200,000 (17)**	**14,800,000 (7)**	**7,400,000**	**4,910,000**
**O-Cessation services**	2007–2010	2 (10)	22,000,000 (9)	1,370,000 (34)	683,000 (34)	448,000 (28)
	2010–2014	5 (25)	68,900,000 (30)	1,950,000 (48)	976,000 (48)	885,000 (56)
	2014–2016	3 (15)	110,000,000 (47)	810 (< 1)	405 (< 1)	405 (< 1)
	2016–2020	10 (50)	31,800,000 (14)	718,000 (18)	359,000 (18)	259,000 (16)
	**Total:2007–2020**	**20 (15)**	**233,000,000 (19)**	**4,040,000 (2)**	**2,020,000**	**1,590,000**
**W-Health warning**	2007–2010	6 (7)	51,000,000 (10)	3,530,000 (8)	1,770,000 (9)	1,150,000 (8)
	2010–2014	25 (28)	101,200,000 (20)	5,830,000 (14)	2,920,000 (14)	1,920,000 (13)
	2014–2016	34 (38)	254,000,000 (50)	26,600,000 (64)	13,300,000 (64)	9,580,000 (64)
	2016–2020	24 (27)	102,000,000 (20)	5,600,000 (13)	2,800,000 (13)	2,320,000 (15)
	**Total: 2007–2020**	**89 (65)**	**507,800,000 (41)**	**41,600,000 (8)**	**20,800,000**	**15,000,000**
**E-Bans on TAPS**	2007–2010	3 (7)	5,600,000 (3)	542,000 (4)	271,000 (4)	176,000 (4)
	2010–2014	19 (42)	116,000,000 (70)	9,690,000 (78)	4,850,000 (78)	3,230,000 (78)
	2014–2016	7 (16)	13,800,000 (8)	868,000 (7)	434,000 (7)	286,000 (7)
	2016–2020	16 (36)	31,300,000 (19)	1,310,000 (11)	653,000 (11)	442,000 (11)
	**Total: 2007–2020**	**45 (33)**	**166,700,000 (14)**	**12,400,000 (7)**	**6,200,000**	**4,130,000**
**R-Raising cigarette taxes**	2007–2010	6 (23)	29,400,000 (28)	4,890,000 (60)	2,450,000 (60)	1,630,000 (59)
	2010–2014	4 (15)	5,700,000 (5)	1,460,000 (18)	728,000 (18)	473,000 (17)
	2014–2016	2 (8)	7,300,000 (7)	1,210,000 (15)	604,000 (15)	393,000 (14)
	2016–2020	14 (54)	63,000,000 (60)	615,000 (8)	308,000 (8)	241,000 (9)
	**Total: 2007–2020**	**26 (19)**	**105,500,000 (9)**	**8,170,000 (8)**	**4,090,000**	**2,740,000**
**All policies**	2007–2010^2^	29 (21)	193,500,000 (16)	13,700,000 (17)	6,860,000 (17)	4,600,000 (16)
	2010–2014^2^	56 (41)	395,700,000 (32)	29,300,000 (36)	14,700,000 (36)	9,880,000 (35)
	2014–2016^2^	43 (32)	398,800,000 (33)	29,400,000 (36)	14,700,000 (36)	10,500,000 (37)
	2016–2020^2^	58 (43)	236,000,000 (19)	8,570,000 (11)	4,280,000 (11)	3,370,000 (12)
	**Total: 2007–2020**^**2**^	**136**	**1,224,000,000**	**81,000,000 (7)**	**40,500,000**	**28,300,000**

Results may differ to the past evaluation as countries (as at 2020) were excluded if they were no longer at the highest level; countries which have achieved the highest level twice were only included the second time the highest level was achieved to avoid double counting

^1^Totals include countries that implement multiple policies

^2^Total is the number of individual countries achieving one or multiple MPOWER policies at the highest level for this time period

^3^Rounding has been applied to all estimates as in Levy et al. [37]

^4^The High Income Country (HIC) risks apply the 0.5 multiplier to the reduction in total number of smokers in all countries to reflect that 50% of all people who smoke will die from smoking. This column applies the HIC multiplier to all countries

^5^The Low and Middle Income Country (LMIC) adjusted estimates apply the 0.65 multiplier to LMICs countries to reflect the lower mortality risk of smoking in these countries. This column applies the risk of death according to a country’s income level (HIC or LMIC)

The MPOWER policies reached over 1.2 billion smokers and their implementation and maintenance was associated with a total reduction in 81.0 million smokers and 28.3 million SADs between 2007 and 2020 (Table 3). Numbers of SADs were higher in each successive evaluation period but decreased in 2020. The reduction in SADs mostly occurred when countries adopted these policies between 2010 and 2016, with the highest number of SADs being reported between 2014 and 2016. The adoption of the health warnings (W) policy accounted for the highest reduction in SADs (53%) with the majority estimated from countries implementing this policy between 2014 and 2016. The adoption of smoke-free air laws (P) (17%), bans on TAPS (E) (15%) and cessation services (O) (6%) have had lesser impact on the reduction in SADs with their impact mostly associated with policy adoption between 2010 to 2014. Half the O policies were adopted between 2016 to 2020. Raising taxes (R) accounted for 10% of the reduction in all SADs mostly averted in the first evaluation period (2007–2010).

### Impact of countries achieving the highest-level MPOWER policies: 2007–2020 by country level income classification

A total of 51 countries (28%) who have signed the FCTC agreement have not implemented any MPOWER policies. Of those countries that have signed the agreement, 72% have implemented and maintained at least one MPOWER policies at best practice. Percentages varied by country income classification, with 16% of the 136 countries classified as LIC, 53% as MIC and 31% as HIC (Table 4). Similar proportions were found for the maintenance of all MPOWER policies by country income classification between 2007 and 2020 (Table 4). There was considerable country level variation within the income categories in relation to the uptake and maintenance of MPOWER policies. A greater proportion of LIC implemented and maintained the P (41%) and E (38%) policies compared to 13% and 15% reported for HIC. A greater proportion of HIC implemented and maintained O and W policies when compared to the other two categories, although this figure for the O policy was still low (23%). A similar low proportion of HIC (17%) and MIC (16%) implemented and maintained the R policy.

**Table 4:** Total and number of countries, policies implemented and maintained, number of smokers affected, reduction in smokers, reach and influence of the policy, and smoking attributable deaths (SADs -LMICs adjusted) by country level income status (LICs = Low Income Countries, MICs = Middle Income Countries and HICs = High Income Countries) for those countries reaching the highest level of MPOWER policies: 2007–2020^1^

MPOWER Policy	Variable	Total n (column %)	LICs n (Row %)	MICs n (Row %)	HICs n (Row %)
Total	Countries	195	35 (18)	105 (54)	55 (28)
Total	Countries signed FCTC	187 (96)	32 (17)	102 (55)	53 (28)
Total	Countries ratified FCTC	181 (93) ^2^	32 (18)	98 (54)	51 (28)
Total	Countries implementing atleast on policy ^3,4^	136 (72)	22 (16)	72 (53)	42 (31)
Total	Policies	233	37 (16)	127 (55)	69 (30)
Total	Number of smokers affected	1,224,080,000	94,333,000 (8)	836,878,000 (68)	292,869,000 (24)
Total	Reduction in smokers	80,998,000	4,128,000 (5)	65,402,000 (81)	11,467,000 (14)
Total	Reach and influence ^5^	6.6%	4.4%	7.8%	3.9%
Total	SADs	28,300,000	1,342,000 (5)	21,255,000 (75)	5,740,000 (20)
P	Countries ^6^	28%	41%	32%	13%
P	Policies	53 (23)	13 (25)	33 (62)	7 (13)
P	Number of smokers affected	211,151,000 (17)	39,913,000 (19)	155,167,000 (73)	16,071,000 (8)
P	Reduction in smokers	14,803,000 (18)	427,000 (3)	13,818,000 (93)	558,000 (4)
P	Reach and influence ^5^	7.0%	1.1%	8.9%	3.5%
P	SADs	4,910,000 (17)	139,000 (3)	4,490,000 (91)	279,000 (6)
O	Countries ^6^	11%	0%	8%	23%
O	Policies	20 (9)	0 (0)	8 (40)	12 (60)
O	Number of smokers affected	232,945,000 (19)	0 (0)	164,439,000 (71)	68,507,000 (29)
O	Reduction in smokers	4,038,000 (5)	0 (0)	2,439,000 (60)	1,599,000 (40)
O	Reach and influence ^5^	1.7%	0%	1.5%	2.3%
O	SADs	1,590,000 (6)	0 (0)	793,000 (50)	799,000 (50)
W	Countries ^6^	48%	34%	44%	62%
W	Policies	89 (38)	11 (12)	45 (51)	33 (37)
W	Number of smokers affected	507,762,000 (41)	29,293,000 (6)	301,213,000 (59)	177,256,000 (35)
W	Reduction in smokers	41,581,000 (51)	2,065,000 (5)	31,221,000 (75)	8,295,000 (20)
W	Reach and influence ^5^	8.2%	7.0%	10.4%	4.7%
W	SADs	15,000,000 (53)	671,000 (4)	10,147,000 (68)	4,150,000 (28)
E	Countries ^6^	24%	38%	25%	15%
E	Policies	45 (19)	12 (27)	25 (56)	8 (18)
E	Number of smokers affected	166,744,000 (14)	23,332,000 (14)	124,964,000 (75)	18,447,000 (11)
E	Reduction in smokers	12,406,000 (15)	1,434,000 (12)	10,414,000 (84)	557,000 (4)
E	Reach and influence ^5^	7.4%	6.1%	8.3%	3.0%
E	SADs	4,130,000 (15)	466,000 (11)	3,385,000 (82)	279,000 (7)
R	Countries ^6^	14%	3.0%	16%	17%
R	Policies	26 (11)	1 (4)	16 (62)	9 (35)
R	Number of smokers affected	105,478,000 (9)	1,795,000 (2)	91,095,000 (86)	12,588,000 (12)
R	Reduction in smokers	8,171,000 (10)	203,000 (2)	7,510,000 (92)	458,000 (6)
R	Reach and influence ^5^	7.7%	11.3%	8.2%	3.6%
R	SADs	2,740,000 (10)	66,000 (2)	2,440,000 (89)	229,000 (8)

^1^Rounding has been applied to all estimates as in Levy et al. [37]

^2^This total includes Andorra which acceded the convention on 11 May 2020, excludes the European Union

^3^These totals exclude those countries that achieved best practice in the M policy only

^4^The denominator is countries that have signed the FCTC, as Argentina, the United States of America, and Morocco who have not ratified the FCTC are included in the WHO reporting

^5^Reach and Influence is the percentage ratio or crude rate per 100 smokers between the reduction in smoker and smokers affected

^6^The denominator is countries that have signed the FCTC and the resulting percentages are column percentage across each column rather than row percentage

Of all MPOWER policies implemented and maintained, MIC accounted for 55%, due to the greater number of countries, and the policies chosen were mostly W and P. Their greater population sizes meant a greater reach with 68% of the total number of smokers affected, globally. These policies accounted for 81% of the total reduction in smokers and 75% of the total reduction in SADs. MIC accounted for a high proportion of adoption and maintenance across individual policies with 62% of all P and R policies and 56% of E policy adopted and maintained, worldwide. These policies impacted a high proportion of total smokers, and in turn a higher reduction in smokers and SADs averted as (Table 4). MIC had lower proportions of O and W policies maintained but had a greater reach in terms of the number of smokers affected and the total reduction in smokers. For these policies, this reach culminated in 50% and 68% of the SADS averted from the O and W policies in MIC, respectively.

Thirty percent of the total MPOWER policies adopted and maintained were in HIC and their implementation resulted in 20% of all SADs averted worldwide. The W policy was the primary policy maintained, followed by the O policy. These policies accounted for 28% and 50% of SADs averted from these policies, respectively. The highest number of countries to implement and maintain O policies were HIC, with half attaining best practice in 2016 to 2020.

LIC implemented and maintained 16% of all MPOWER policies which resulted in averting 5% of SADs, worldwide. LIC accounted for 25% and 27% of all P and E policies, which contributed to 3% and 11% of SADs averted by these policies, respectively. No LIC have adopted and maintained the O policy (Cessation services) and only one (Madagascar in 2010) has adopted and maintained raising cigarette taxes (R) to the highest level (Table 4).

The reach and influence of the adoption and maintenance of MPOWER policies by country level income status over 2007 to 2020 can be demonstrated by comparing the ratio of the reduction in smokers to total number of smokers affected in Table 4 (Reach and influence). Overall, the model estimated that reach and influence of the MPOWER policies resulted in a reduction in 6.6 smokers per 100 smokers globally. This equated to a reduction per 100 smokers of 4.4 smokers in LIC, 7.8 smokers in MIC and 3.9 smokers in HIC. In LIC, there were no O policies currently at best practice, and the P policy had the lowest reach and influence with a reduction of 1 smoker per 100 smokers. Conversely, the W policy resulted in a reduction of 7 smokers per 100 smokers. Results for the R policy although higher should be treated as illustrative only as it represents only one LIC. For MIC, the W policy had the greatest reduction of 10 smokers per 100 smokers. With the exception of O policies (1.5 per 100 smokers), the other policies all had a reduction of around 8 smokers per 100 smokers. The reduction for HIC were consistently lower, with all policies reducing 2–3 smokers per 100 smokers, apart from the W policy which reduced 4.7 smokers per 100 smokers.

## Discussion

This study was able to accurately replicate the Abridged SimSmoke model [36, 37] using the published method with a number of adaptions where documentation was lacking. Its application with up-to-date data showed the progress of recent policy implementation at best practice globally. The model estimated that between 2016 and 2020 there was 8.57 million fewer smokers and a reduction of 3.37 million SADs. Augmenting this data with past evaluations [34–36] suggests there has been a reduction of 81.0 million smokers and 28.3 million SADs since the inception of the MPOWER policies in 2007 through to 2020. Further analysis of these results by country income level showed variations in implementation, maintenance, reach and influence of these policies highlighting inequity in their implementation and maintenance globally. These results support previously published data suggesting the progress in the implementation of the MPOWER remains uneven across countries and policy domains [16].

In replicating the Abridged SimSmoke model we followed the documented process [36, 37] as best as possible. For some policies there was inadequate documentation to execute the published methodology and we had to apply our own methods to allocate effect sizes. For example, it was not clear how Levy et al. [36, 37] allocated effect sizes for banning smoking (P) in indoor workplaces and the provision of treatment services (O) in sectors. Here, we took a conservative approach to proportionally allocate the specific effect size based on the number of places where bans were placed or where services were available. There also were discrepancies between the text and the mathematical equations presented in the supplementary material [37] meaning we had to decide which adjustors to include in our analysis. We chose, as Levy had done in a previous study [45], to include a country level labour force adjustor in the P equation with the rationale that it reflected that smoke-free work site laws primarily influence the population who work indoors. We also chose to include a publicity adjuster in the equation for the O policy to reflect program uptake. These assumptions likely underestimated the country level effect sizes for P and O policies and some caution should be taken when comparing these estimates over time. The validation step replicated precise results for 41 out of the 56 countries, and for countries implementing W and R policies. Where differences in SADs were apparent, the majority of differences were small. These small discrepancies were due to differences in input data such as country populations and adjustment values such as the level of urbanisation. There were, however, a few exceptions. The largest discrepancy between results was for SADs estimated for cessation services (O policy) which reflected a difference in the WHO monitoring data i.e. a quit line in India was included in our analysis which was not included in the published results [36, 37]. Additionally, there were also discrepancies found for Romania and Afghanistan (P) and Afghanistan and Senegal (E). These discrepancies may also be due to reporting discrepancies as the WHO continually re-evaluate and update their reporting, i.e. the review of the 2014 [41] and 2018 [4] data report that 3% of data points were corrected. Precisely estimating SADs for a high number of countries, reporting small differences in others and finding underestimation in only a small number of individual countries rather than across all countries within a MPOWER category demonstrates that we have been able to replicate the model robustly.

Our updated analysis of the policies implemented between 2016 and 2020 showed that, similar to the previous evaluation [36], the largest number of smokers affected, reduction in smokers and averted SADs was from the adoption of health warnings (W) at the highest level (102 million, 5.6 million smokers and 2.3 million SADs, respectively). Two countries (United States and Pakistan) accounted for most of this reduction with a one-step strengthening of the policy to best practice demonstrating the benefit of making this final change in populous countries. These results provide further evidence of the success of this policy implementation globally [46] and while there are numerous reasons of why warnings have been so successful, one of note is that the cost of warnings is paid by industry which is attractive to all governments [45]. It should also be noted that while a larger number of countries have achieved this level, the increased SADs is also due to an increase in the effect sizes applied to model for this policy for the periods after 2014 [36]. In comparison, other policies had smaller influence. For example, P and E policies were estimated to affect over 7.7 million and 31.3 million smokers but only resulted in small reductions in SADs, 106,000 and 442,000 SADs respectively. This was due to low country level compliance, the average compliance values were 3.9 (std. dev. = 1.5) and 3.4 (std. dev. = 2.6) out of 10 for P and E policies (data not shown). This highlights that implementation of P and E policies requires more than just passing laws and bans, they require a high degree of country level compliance to achieve better protection. A global study [17] found that over time there have been minimal increases in compliance for P policies, especially in LIC and MIC. Higher compliance has been found to be larger for direct advertising, but since 2011 compliance with promotion and sponsorship restrictions have not increased. It is also difficult to secure funding to maintain a high level of compliance after laws have been implemented or to cover costs for cessation treatment (O policies), particularly in LIC and MICs where resources are limited. This may explain why no LIC has achieved best practice in the O policy within the 2016 to 2020 analysis, instead this policy is more recently dominated by HICs and MICs. These resource issues emphasise the importance of establishing and raising cigarette taxes which are one way to secure sustainable funding [47], however they must be significant enough to reduce tobacco use [48] and not just a revenue raising tool.

The MPOWER package has had large variations in its implementation and maintenance, and reach and influence, creating significant inequality in the benefits of the policies globally. The sheer number and population sizes of the MIC meant that they accounted for a high proportion of reported benefits. The W policy had the biggest reduction in SADs over all country income groups and the largest uptake in MIC and HIC. We found that the uptake and maintenance of at least one policy occurred in 69% (22 of 32 countries see Table 4), 70%, and 79% of all the LIC, MIC and HIC that have signed the FCTC, respectively. This equated to around 30% of countries within each income category having implemented and maintained one policy, around 30% also maintained two policies and around 10% of countries maintained three policies (data not shown). For some specific policies, the lack of implementation progress may reflect a lack of resources to achieve best practice but also challenges the ‘one size fits all’ approach of the MPOWER package [25]. Here, different policies may be more effective in different areas of the world [21, 49]. Therefore it is important to consider each country’s stage in the smoking epidemic [50] as well as their economic, cultural and political determinants [25, 51] in the progress and overall evaluation of the impact of MPOWER policies. This is perhaps evident in the uptake of P policies where less than half of all LIC have implemented them and when implemented the overall influence of the policy was low, reaching one in 100 smokers. So even when these policies are implemented at best practice, the contextual factors of the country that are considered in the model, such as low levels of urbanisation and high levels of unemployment will influence the estimated policy impact. The reverse can also be seen for HIC implementing the O policy where within the model, urbanisation and publicity adjustors are higher than that for MIC and LIC meaning that a greater effect size will be allocated. Given the influence of these inherent country level factors, it is therefore critical that countries place added effort into increasing the publicity and compliance of these policies in order to amplify the benefits of their implementation [17].

Our analysis has again demonstrated the lack of continued implementation of MPOWER policies in LIC and MIC countries [21]. However, the lack of uptake of MPOWER policies by HIC is also concerning, especially the P, E and R policies where less than 20% of all HIC have implemented and maintained them. While we can attribute this slow implementation to ongoing influence and legal challenges undertaken by the tobacco industry [6, 16], less is known about why as a category HIC have not achieved swifter best practice implementation given their access to additional resources. Also concerning is that the range of influence of these policies is also lower in comparison to MIC. Further investigation is needed to explore why this has occurred.

The insight that has been established on the effectiveness of the MPOWER package is dependent on the strengths and limitations of the Abridged SimSmoke model. These have been noted in detail in another evaluation [36]. Strengths include the use of WHO monitoring data of policy changes and the effectiveness of policy changes in the Abridged SimSmoke model based on an extensively validated statistical model. Aside from the methodological issues in replicating some parts of the model, there are several considerations when evaluating the O and R policy components in countries that reach best practice. Like others [36], we also found in the 2016 to 2020 analysis that three countries had no effect size for the O policy. While the WHO report that these countries have achieved best practice, the increment to best practice was achieved through some cessation services being cost covered. The replicated model only considers availability and not cost coverage and therefore no effect size is given, and as a result no SADs estimated. Tobacco taxation is also seen as an effective strategy because of its potentially large impact and low implementation cost [24]. However, for those countries achieving the highest level of the R policy, its effectiveness estimated by the model is impacted, sometimes adversely, by the rate of inflation. For example, in the 2016 to 2020 analysis, there was high inflation in the highly populated countries of Brazil and Thailand and because of this, the model estimated no SADs from this policy for these countries. As a result, the model estimated that globally there was only a 1% reduction in the number of smokers from a potential of 63 million smokers affected in this time period. Further considerations need to be given to the methodological aspects of the model, in particularly for the R policy as both price and income growth determine affordability of a product [24] and this information and others may need to be included in the model, in future.

The model was development to estimate the number of SADs averted since the inception of the FCTC and MPOWER package using change from baseline (2007 WHO reporting) as an evaluation measure. Countries starting from a low baseline have the greatest potential to change and conversely, those who met best practice before 2007 are not considered within this model. For example, best practice was reported for Brazil in 2007, therefore no SADs were attributed to O and W policies, and as such the results don’t truly reflect their overall tobacco control efforts to date. A second consideration is that the application of the model does not capture incremental implementation of policies prior to the point of achieving best practice. A country may incrementally move over time to a score of 4 and then later achieves best practice (a score of 5). The model only considers the movement from 4 to 5, making no allowances for past incremental increases and therefore underestimating their total outcomes in tobacco control progression. This may explain why we had lower estimated total SADs in the 2016 to 2020 period compared to earlier evaluation periods and this will likely be the case in future evaluations of these policies. Finally, partial implementation of polices are also not captured in this model thus excluding the benefits of adopted policies that are not reaching the level of best practice. If this was to be included in future models, we must consider if the effect of policy implementation at the highest level could be non-linear which may have a greater or lower marginal impact, compared with an implementation at a lower level [23]. While the highest level of achievement must still be the aim, some countries may never achieve this, but their partial progress should still be evaluated. Finally, the model is focussed on the effects of changes in singular policies and these changes are treated as independent of one another. The model therefore does not consider the effect on SAD’s when policies work in conjunction with each other [52]. Recent analysis has shown synergies between policies [25, 53]. For example, implementation of P and O policies together reported a 39% increase in cessation rate while combining P, W and E policies decreased the adolescent smoking prevalence rate by 26% [31]. In the future, the application of this specific model may become less relevant and modifications which consider these considerations may be more of interest.

## Conclusion

Considerable progress has been made in reducing the prevalence of smokers globally, with the FCTC and MPOWER policy package playing a significant role. However, when comparing the progress by country income we found inequality in their implementation and maintenance, reach and influence, and the number of SADs averted. Continued efforts are required to strengthen policies in these and other areas to further reduce the prevalence of smoking. Future research to modify the evaluation model could provide a more comprehensive assessment of tobacco control by considering partial implementation, the time to achieving best practice, the synergies between policies, and ongoing efforts to implement and maintain MPOWER policies.

## Data Availability

The data that support the findings of this study are available from the WHO.

## Appendix 1

**Table 5:** Policy specifications and effect sizes used in the replication of the Abridged SimSmoke model

Policy	Description	Short-term effect size (% effect)^a^	Long-term multiplier	Awareness adjustor^b^	Urban Adjustor^c^	Labour Adjustor ^d^	Lower and upper bounds
**Protecting (P): Smoke-free air laws (effects are additive over policies)**							
Indoor workplaces: smoke free	Ban in all indoor workplaces	6% distributed by a proportional allocation	1.25	1.5	yes	yes	(-50%, + 50%)
Restaurants: smoke free	Ban in all indoor restaurants	2%	1.25	1.5	yes	yes	(-50%, + 50%)
Pubs and bars: smoke free	Ban in all indoor pubs and bars	1%	1.25	1.5	yes	yes	(-50%, + 50%)
Other indoor places	Ban in all other public places	1%	1.25	1.5	yes	yes	(-50%, + 50%)
Compliance	Ranking out of 10 scaled to continuous value between 0 and 1 and averaged over the two time periods	25% of the effect depends on the ratio of compliance					
Publicity	Based on tobacco control funding (US$) per capita. Set at high (1), medium (0.75) and low (0.5)	25% of effect depends on publicity					
**Offering (O): Cessation services (effects are additive over policies)**							
Quit line	If available. Quit line assumed operating active	0.75%	2.5	1.5	yes	no	(-50%, + 50%)
Availability of Nicotine Replacement Therapy (NRT)	If NRT provided by general store or pharmacy w/ Rx = 1 If NRT is provided by general store or pharmacy (no Rx required) = 2	1% if score of 2	2.5	1.5	yes	no	(-50%, + 50%)
Provision of treatments	By types of facility: Available in some or most facilities and cost covered as either partial or fully to attain the effect size	2.25% distributed by a proportional allocation	2.5	1.5	yes	no	(-50%, + 50%)
Publicity	Based on tobacco control funding ($) per capita. Set at high (1), medium (0.75) andlow (0.5)	Set in US dollars and 15 years plus population					
**Warning (W): Health warnings (first four categories are mutually exclusive)**							
Complete health warnings	Large warnings with all appropriate characteristics. Score = 5	4.0%	2	2	no	no	(-50%, + 50%)
Strong health warnings	Medium size warnings with all appropriate characteristics or large warnings missing some appropriate characteristics. Score = 4	2.0%	2	2	no	no	(-50%, + 50%)
Medium health warnings	Medium size warnings missing some or many appropriate characteristics or large warnings missing many appropriate characteristics. Score = 3	1.0%	2	2	no	no	(-50%, + 50%)
Low health warnings	No warning or small warnings. Score = 2	0%	2	2	no	no	(-50%, + 50%)
**Enforcing (E): Bans on tobacco advertising, promotion and sponsorship (TAPS) (first four categories are mutually exclusive)**							
Complete ban on TAPS	Ban on all forms of direct and/or indirect advertising (or at least 90% of the population covered by complete subnational bans). Score = 5	5%	1.3	2	no	no	(-50%, + 50%)
Strong ban on TAPS	Ban on national television, radio and print media as well as on some but not all other forms of direct and/or indirect advertising. Score = 4	3%	1.3	2	no	no	(-50%, + 50%)
Medium ban on TAPS	Ban on national television, radio and print media only. Score = 3	1%	1.3	2	no	no	(-50%, + 50%)
Absence of ban on TAPS	Complete absence of ban, or ban that does not cover national television, radio and print media, Score = 2	0%	1.3	2	no	no	
Compliance	Ranking out of 10 standardised to a continuous value between 0 and 1 and averaged over the two time periods	50% of the effect depends on the ratio of compliance			no	no	
**Raising (R): Raise cigarette taxes**							
Increase in retail price of cigarettes due to taxes	Cigarette price in local currency and adjusted by inflation. Smoking prevalence price elasticities applied to % change in inflation-adjusted price	Price elasticities based HICs^e^ (0.15) or LMICs^e^ (0.20)	2	no	no	no	(-50%, + 50%)

Country inflation rates taken from https://data.worldbank.org/indicator/FP.CPI.TOTL.ZG. Accessed September 2023

Country rate of employment in agriculture taken from https://data.worldbank.org/indicator/SL.GR.EMPL.ZS. Accessed September 2023

Coutes taken from https://data.worldbank.org/indicator/SL.UEM.TOTL.NE.ZS. Accessed September 2023

Country income status taken from https://datahelpdesk.worldbank.org/knowledgebase/articles/906519-world-bank-country-and-lending-groups. Accessed September 2023

^a^The initial effect size is the short term-effect that is multiplied by the long-term multiplier with Awareness, Urban and Labour adjustments as specified in the table

^b^The Awareness adjustor is multiplied by the effect size for low and middle-income countries

^c^The Urban adjustor reduces the effect to reflect the percent urban for the policies indicated

^d^The Labour Adjustor reduces the effect to reflect the percent working for the policies indicated

^e^*HIC* High-Income Country, *L**MIC* Low and Middle Income Country

## Appendix 2

**Table 6:** Reduction in smoking-attributable deaths (SADs) from countries achieving the highest MPOWER level for smoke-free air laws (P): 2016–2020^1^

Intervention country	Income status	Smoking rate		Total no. of smokers affected	Long-term effect size (%)	Total reduction in no. of smokers (row %)	Reduction in smoking-attributable deaths	
		**Males (%)**	**Females (%)**					
							**HIC risks**^**2**^** (column %)**	**LMIC-adjusted**^**3**^** (column %)**
1. Antigua and Barbuda	HIC	4.4	2.2	2,430	-5.6	136 (6)	68 (< 1)	68 (< 1)
2. Benin	LIC	8.7	1.3	327,000	-2.6	8,470 (3)	4,230 (3)	2,750 (3)
3. Bolivia	MIC	22.1	5.6	1,080,000	-5.0	54,000 (5)	27,000 (17)	17,500 (17)
4. Burundi	LIC	15.8	2.1	536,000	-1.4	7,490 (1)	3,750 (2)	2,440 (2)
5. Ethiopia	LIC	6.5	0.9	2,380,000	-2.5	59,500 (3)	29,700 (18)	19,300 (18)
6. Guyana	MIC	23.2	2.5	71,700	-5.4	3,890 (5)	1,940 (1)	1,260 (1)
7. Jordan	MIC	57.1	12.9	2,310,000	-5.5	127,000 (5)	63,400 (39)	41,200 (39)
8. Niue	LIC	20.5	11.0	190	-8.6	17 (9)	8 (< 1)	5 (< 1)
9. Paraguay	MIC	19.8	4.8	609,000	-6.7	40,700 (7)	20,300 (12)	13,200 (12)
10. Saint Lucia	MIC	14.3	1.8	11,700	-5.6	655 (6)	327 (< 1)	213 (< 1)
11.Tajikistan	LIC	14.7	0.3	430,000	-5.5	23,500 (5)	11,700 (7)	7,630 (7)
**Total**				**7,760,000**		**325,000 (4)**	**163,000**	**106,000**

Smoking rates for Antigua and Barbuda were taken from https://files.tobaccoatlas.org/wp-content/uploads/pdf/antigua-and-barbuda-country-facts-en.pdf; Niue from https://gsthr.org/countries/profile/niu/; Saint Lucia from-https://files.tobaccoatlas.org/wp-content/uploads/pdf/st-lucia-country-facts-en.pdf; and Tajikistan from https://documents1.worldbank.org/curated/pt/357221561130314918/pdf/Tajikistan-Overview-of-Tobacco-Use-Tobacco-Control-Legislation-and-Taxation.pdf. Accessed September 2023

^1^Rounding has been applied to all estimates as in Levy et al. [37]. Numbers may not sum due to rounding

^2^The High Income Country (HIC) risks apply the 0.5 multiplier to the total reduction in number of smokers in all countries to reflect that 50% of all people who smoke will die from smoking

^3^The Low and Middle Income Country (LMIC) adjusted estimates apply the 0.65 multiplier to the total reduction in number of smokers in LMICs countries to reflect the lower mortality risk of smoking in these countries

## Appendix 3

**Table 7:** Reduction in smoking-attributable deaths (SADs) from countries achieving the highest MPOWER level for cessation services (O): 2016–2020.^1^

Intervention country	Income status	Smoking rate		Total no. of smokers affected	Long term effect size (%)	Total reduction in no. of smokers (row %)	Reduction in smoking attributable deaths	
		**Males (%)**	**Females(%)**					
							**HIC risks**^**2**^** (column %)**	**LMIC-adjusted**^**3**^** (column %)**
1. Austria	HIC	28.9	25.8	2,080,000	0.0	0	0	0
2. Cook Islands	HIC	28.7	21.1	3,250	-1.3	43 (1)	22 (< 1)	22 (< 1)
3. Costa Rica	MIC	13.6	4.8	361,000	-5.7	20,700 (6)	10,400 (3)	6,740 (3)
4. Czechia	HIC	35.4	26.7	2,790,000	-2.6	71,100 (3)	35,500 (10)	35,500 (14)
5. Jordan	MIC	57.1	12.9	2,310,000	-1.4	31,600 (1)	15,800 (4)	10,300 (4)
6. Philippines	MIC	40.6	6.8	17,400,000	-3.0	519,000 (3)	259,000 (72)	169,000 (65)
7. Saudi Arabia	HIC	25.6	2.0	4,070,000	-1.4	55,800 (1)	27,900 (8)	27,900 (11)
8. Slovakia	HIC	38.3	26.0	1,470,000	-1.4	20,200 (1)	10,100 (3)	10,100 (4)
9. Sweden	HIC	16.7	15.6	1,330,000	0.0	0	0	0
10. Tonga	MIC	47.7	15.4	20,800	0.0	0	0	0
				**31,830,000**		**718,000 (2)**	**360,000**	**260,000**

^1^Rounding has been applied to all estimates as in Levy et al.[37]. Numbers may not sum due to rounding

^2^The High Income Country (HIC) risks apply the 0.5 multiplier to the total reduction in number of smokers in all countries to reflect that 50% of all people who smoke will die from smoking

^3^The Low and Middle Income Country (LMIC) adjusted estimates apply the 0.65 multiplier to the total reduction in number of smokers in LMICs countries to reflect the lower mortality risk of smoking in these countries

## Appendix 4

**Table 8:** Reduction in smoking-attributable deaths (SADs) from countries achieving the highest MPOWER level for health warnings (W): 2016–2020.^1^

Intervention country	Income status	Smoking rate		Total no. of smokers affected	Long term effect size (%)	Total reduction in no. of smokers (row %)	Reduction in smoking attributable deaths	
		**Males (%)**	**Females (%)**					
							**HIC risks**^**2**^** (column %)**	**LMIC adjusted**^**3**^** (column %)**
1. Barbados	HIC	12.8	2.0	17,000	-8.0	1,360 (8)	679 (< 1)	679 (< 1)
2. Cameroon	MIC	12.2	0.4	906,000	-8.0	72,500 (8)	36,200 (1)	23,600 (1)
3. Croatia	HIC	38.3	35.9	1,320,000	-6.0	79,000 (6)	39,500 (1)	39,500(2)
4. Cyprus	HIC	48.3	23.5	355,000	-6.0	21,300 (6)	10,600 (< 1)	10,600 (< 1)
5. Ethiopia	LIC	6.5	0.9	2,380,000	-12.0	285,000 (12)	143,000 (5)	92,800 (4)
6. Gambia	LIC	21.5	0.5	137,000	-12.0	16,400 (12)	8,210 (< 1)	5,340 (< 1)
7. Georgia	MIC	56.9	7.1	973,000	-12.0	117,000 (12)	58,400 (2)	38,000 (2)
8. Ghana	MIC	5.1	0.3	506,000	-8.0	40,400 (8)	20,200 (1)	13,100 (1)
9. Guyana	MIC	23.2	2.5	71,700	-16.0	11,500 (16)	5,740 (0)	3,730 (0)
10. Honduras	MIC	33.0	2.0	1,130,000	-8.0	90,700 (8)	45,300 (2)	29,500 (1)
11. Luxembourg	HIC	23.2	20.3	111,000	-6.0	6,640 (6)	3,320 (< 1)	3,320 (< 1)
12. Mauritania	MIC	15.7	2.6	241,000	-16.0	38,500 (16)	19,200 (1)	12,500 (1)
13. Montenegro	MIC	33.5	35.8	178,000	-8.0	14,200 (8)	7,120 (< 1)	4,630 (< 1)
14. Niger	LIC	13.8	0.1	774,000	-8.0	61,900 (8)	30,900 (1)	20,100 (1)
15. Nigeria	MIC	6.4	0.4	3,760,000	-8.0	301,000 (8)	150,000 (5)	97,700 (4)
16. Pakistan	MIC	25.4	3.5	20,200,000	-8.0	1,620,000 (8)	809,000 (29)	526,000 (23)
17. Qatar	HIC	21.7	2.1	425,000	-4.0	17,000 (4)	8,490 (< 1)	8,490 (< 1)
18. Saint Lucia	MIC	14.3	1.8	11,700	-16.0	1,880 (16)	939 (< 1)	610 (< 1)
19. Saudi Arabia	HIC	25.6	2.0	4,070,000	-4.0	163,000 (4)	81,400 (3)	81,400 (4)
20. Slovenia	HIC	24.9	19.9	395,000	-6.0	23,700 (6)	11,900 (< 1)	11,900 (1)
21. Spain	HIC	29.5	27.2	11,300,000	-4.0	451,000 (4)	226,000 (8)	226,000 (10)
22. Tajikistan	LIC	14.7	0.3	430,000	-16.0	68,800 (16)	34,400 (1)	22,400 (1)
23. Timor-Leste	MIC	59.3	5.5	257,000	-8.0	20,600 (8)	10,300 (< 1)	6,680 (< 1)
24. United States of America	HIC	22.9	16.4	52,100,000	-4.0	2,080,000 (4)	1,040,000 (37)	1,040,000 (45)
**Total**				**102,000,000**		**5,600,000 (5)**	**2,800,000**	**2,320,000**

Smoking rates for Honduras were taken from https://gsthr.org/countries/profile/hnd/; Saint Lucia-from https://files.tobaccoatlas.org/wp-content/uploads/pdf/st-lucia-country-facts-en.pdf; and Tajikistan from https://documents1.worldbank.org/curated/pt/357221561130314918/pdf/Tajikistan-Overview-of-Tobacco-Use-Tobacco-Control-Legislation-and-Taxation.pdf . Accessed September 2023

^1^Rounding has been applied to all estimates as in Levy et al. [37]. Numbers may not sum due to rounding

^2^The High Income Country (HIC) risks apply the 0.5 multiplier to the total reduction in number of smokers in all countries to reflect that 50% of all people who smoke will die from smoking

^3^The Low and Middle Income Country (LMIC) adjusted estimates apply the 0.65 multiplier to the total reduction in number of smokers in LMICs countries to reflect the lower mortality risk of smoking in these countries

## Appendix 5

**Table 9:** Reduction in smoking-attributable deaths (SADs) from countries achieving the highest MPOWER level for bans on tobacco advertising, promotion, and sponsorship (TAPS) (E): 2016–2020.^1^

Intervention country	Income status	Smoking rate		Total no. of smokers affected	Long term effect size (%)	Total reduction in no. of smokers (row %)	Reduction in smoking attributable deaths	
		**Males (%)**	**Females (%)**					
							**HIC risks **^**2**^** (column %)**	**LMIC-adjusted **^**3**^** (column %)**
1. Algeria	MIC	34.5	0.7	5,220,000	-3.3	170,000 (3)	84,800 (13)	55,100 (12)
2. Antigua and Barbuda	HIC	4.4	2.2	2,430	-3.4	83 (3)	42 (< 1)	42 (< 1)
3. Azerbaijan	MIC	42.3	0.1	1,580,000	-3.5	55,400 (4)	27,700 (4)	18,000 (4)
4. Benin	LIC	8.7	1.3	327,000	-3.8	12,300 (4)	6,160 (1)	4,010 (1)
5. Congo	MIC	27.0	0.9	423,000	-2.7	11,500 (3)	5,770 (1)	3,750 (1)
6. Côte d'Ivoire	MIC	23.2	0.6	1,750,000	-6.5	114,000 (7)	57,000 (9)	37,100 (8)
7. Democratic Republic of the Congo	LIC	22.6	0.7	5,220,000	-3.1	163,000 (3)	81,400 (12)	52,900 (12)
8. Ethiopia	LIC	6.5	0.9	2,380,000	-4.2	99,000 (4)	49,500 (8)	32,200 (7)
9. Guyana	MIC	23.2	2.5	71,700	-8.8	6,290 (9)	3,150 (< 1)	2,040 (< 1)
10. Iraq	MIC	36.7	2.0	4,590,000	-3.4	155,000 (3)	77,600 (12)	50,400 (11)
11. Jordan	MIC	57.1	12.9	2,310,000	-3.4	78,000 (3)	39,000 (6)	25,400 (6)
12. Mauritania	MIC	15.7	2.6	241,000	-6.8	16,400 (7)	8,210 (1)	5,340 (1)
13.Niue	LIC	20.5	11.0	192	-7.8	15 (8)	7 (< 1)	5 (< 1)
14. Saudi Arabia	HIC	25.6	2.0	4,070,000	-2.3	92,600 (2)	46,300 (7)	46,300 (10)
15. Slovenia	HIC	24.9	19.9	395,000	-2.3	8,990 (2)	4,490 (1)	4,490 (1)
16. Venezuela	MIC	16.7	9.9	2,760,000	-11.7	323,000 (12)	162,000 (25)	105,000 (24)
**Total**				**31,340,000**		**1,305,000 (4)**	**653,000**	**442,000**

Smoking rates for Niue from https://gsthr.org/countries/profile/niu/; and Venezuela from https://tobaccoatlas.org/country/venezuela/. Accessed September 2023

^1^Rounding has been applied to all estimates as in Levy et al.[37]. Numbers may not sum due to rounding

^2^The High Income Country (HIC) risks apply the 0.5 multiplier to the total reduction in number of smokers in all countries to reflect that 50% of all people who smoke will die from smoking

^3^The Low and Middle Income Country (LMIC) adjusted estimates apply the 0.65 multiplier to the total reduction in number of smokers in LMICs countries to reflect the lower mortality risk of smoking in these countries

## Appendix 6

**Table 10:** Reduction in smoking-attributable deaths (SADs) from countries reaching the highest MPOWER level for raising taxes (R): 2016–2020.^1^

Intervention country	Income status	Smoking rate		Total no. of smokers affected	Long-term effect size (%)	Total reduction in no. of smokers (row %)	Reduction in smoking attributable deaths	
		**Males (%)**	**Females (%)**					
							HIC risks ^2^ (column %)	LMIC-Adjusted ^3^ (column %)
1. Andorra	HIC	37.2	29.4	24,800	-2.3	570 (2)	290 (< 1)	290 (< 1)
2. Brazil	MIC	16.8	9.8	21,800,000	0.0	0 (0)	0	0
3. Denmark	HIC	18.5	17.8	872,000	-8.8	76,700 (9)	38,400 (12)	38,400 (16)
4. Egypt	MIC	47.6	0.4	15,600,000	-0.03	4,550 (0)	2,275 (1)	1,480 (1)
5. Georgia	MIC	56.9	7.1	973,000	-20.0	195,000 (20)	97,500 (32)	63,200 (26)
6. Mauritius	MIC	38.5	3.1	213,000	-1.8	3,920 (2)	1,960 (1)	1,270 (1)
7. Montenegro	MIC	33.5	35.8	178,000	-10.0	17,800 (10)	8,900 (3)	5,800 (2)
8. Morocco	MIC	26.6	1.0	3,550,000	0.0	0 (0)	0	0
9. Netherlands	HIC	25.0	20.4	3,250,000	-3.5	115,000 (4)	57,500 (19)	57,500 (24)
10. New Zealand	HIC	15.6	12.8	540,000	-7.7	41,500 (8)	20,800 (7)	20,800 (9)
11. North Macedonia	MIC	57.9	39.0	842,000	-12.3	103,000 (12)	51,500 (17)	33,600 (14)
12. Portugal	HIC	30.9	20.1	2,230,000	0.0	0 (0)	0	0
13. Sri Lanka	MIC	25.6	0.3	1,970,000	-2.9	57,300 (3)	28,650 (9)	18,600 (8)
14. Thailand	MIC	38.1	1.6	11,000,000	0.0	0 (0)	0	0
**Total**				**63,100,000**		**615,000 (1)**	**307,500**	**241,000**

Smoking rates for North Macedonia were taken from which was from https://tobacconomics.org/files/research/645/237-fact-sheet-nmk-stc-see-2019-v4-1.pdf. Accessed September 2023

^1^Rounding has been applied to all estimates as in Levy et al.[37]. Numbers may not sum due to rounding

^2^The High Income Country (HIC) risks apply the 0.5 multiplier to the total reduction in number of smokers in all countries to reflect that 50% of all people who smoke will die from smoking

^3^The Low and Middle Income Country (LMIC) adjusted estimates apply the 0.65 multiplier to the total reduction in number of smokers in LMICs countries to reflect the lower mortality risk of smoking in these countries
